# Work-related instant messaging and calling stress (WRIMCS) among physicians: a novel occupational health risk?

**DOI:** 10.1186/s12995-025-00478-1

**Published:** 2025-09-02

**Authors:** Aldo Shpuza, Edlira Bylykbashi, Enver Roshi, Genc Burazeri

**Affiliations:** 1 Department of Public Health, Faculty of Medicine, University of Medicine, Tirana, Albania; 2 Department of Clinical Subjects, Faculty of Technical Medical Sciences, University of Medicine, Tirana, Albania; 3 Department of International Health, Care and Public Health Research Institute, Maastricht University, Maastricht, The Netherlands

**Keywords:** Mediation analysis, Occupational stress, Psycho-physiological stressors, Technostress, Work-Related instant messaging and calling (WRIMC)

## Abstract

**Aim:**

To assess the association between WRIMC and perceived stress among physicians through the lens of ‘occupational context’ and psycho-physiological stressors as mediators.

**Methods:**

A cross-sectional study was conducted in Albania in January 2025 including a representative sample of 367 physicians (≈ 66% females; overall response rate: ≈90%). A structured 36-item questionnaire included demographic characteristics, WRIMC exposure and related psycho-physiological stressors and the Perceived Stress Scale (PSS). Multivariable-adjusted ordinal logistic regression was used to assess the direct association between WRIMC-related exposures and PSS scores, whereas mediation analysis was used to assess indirect effects using the perceived impact of work on stress level and psycho-physiological stressors as mediators.

**Results:**

Physicians had moderate-to-high exposure frequencies to work-related phone usage, instant messages and calling, app-based instant messaging, WRIMC beyond work hours, during vacations or after 22:00 o’clock. WRIMC via apps, irritability due to WRIMC interruptions, guilt over not responding to WRIMC and mental fatigue due to WRIMC were identified as ordinal correlates of perceived stress (β = 0.48, β = 0.65, β = 0.33 and β = 0.37, respectively). The perceived impact of work served as a partial mediator (β = 1.07) between WRIMC and PSS scores, though a direct effect of WRIMC on PSS was also observed (β = 2.57). Physicians’ age was a negative covariate in both indirect and direct effects.

**Conclusions:**

Physicians are highly exposed to WRIMC which may be associated with certain psycho-physiological stressors. The perceived occupational stress context of instant messaging and calling might represent a significant pathway beyond technostress per se, which may be interconnected with or distinct from work-related stress.

**Supplementary Information:**

The online version contains supplementary material available at 10.1186/s12995-025-00478-1.

## Introduction

The American Psychological Association defines work-related stress as the physiological and psychological response to harmful workplace conditions or events that negatively impact health and well-being [1]. Although the concept of stress is complex that varies in duration, sources (stressors) and responses, it is generally classified into subtypes, with its biopsychosocial significance often being confined to acute, traumatic and chronic stress [2, 3]. Different workplace stressors contribute to both acute and chronic stress which eventually may lead to health issues such as fatigue, burnout, and post-traumatic symptoms, particularly among medical professionals [4–6]. Moreover, the stress that arises from using technology—often called techno-stress—has become a notable concern that impacts individuals in both professional and personal spheres, potentially compromising their mental and physical health, job performance, and social interactions [7, 8]. Consistently, research indicates that the use of digital technologies brings specific psychosocial demands, such as higher workload, complexity, and work-family conflicts, leading to psychobiological stress reactions, especially among physicians, in whom medium levels of technostress and its impact on exhaustion and job engagement have been observed [9, 10]. Building on this, the use of mobile phones at work can further impact these psychosocial aspects such as engagement, employee innovation and work-family conflicts [11]. Statistical data show that daily mobile usage typically ranges from 3 to 4 h [12]—suggesting that a significant time may be spent on calls and messaging, potentially fostering continuous engagement in instant communication. Furthermore, a qualitative evidence synthesis showed that the unregulated use of personal phones has become an integral part of healthcare work, which, while improving responsiveness to patients, colleagues, and managers, it can also blur the lines between professional and personal boundaries [13]. While communication through messaging and calling extends beyond working hours, work-family conflicts becomes even more concerning [14]. Additionally, such work-related communication extending beyond working hours increases the odds of anxiety [15]. The implementation of clear and realistic policies regulating smartphone use in healthcare settings has become essential to balance the benefits in communication and clinical decision-making with certain risks such as distraction, depersonalization of care, increased stress, and compromised patient safety [16, 17]. In Albania, professional relationships—whether between doctors and patients, colleagues, or managers—are often marked by unclear personal boundaries, most likely due to the country’s socio-cultural context. From this perspective, the nature of patient-doctor relationships in Albania is frequently shaped by socio-cultural norms that blur the line between professional and personal interactions. These relationships often lack clearly defined boundaries, which can manifest in both subtle and overt ways including instant messaging and calling of doctors even outside working hours. This dynamic is rooted in broader cultural characteristics, including a collectivist ethos, strong interpersonal networks, and a historical reliance on informal systems of trust and reciprocity in the face of institutional deficiencies. Specifically for clinical settings, this blurred boundary may lead to greater emotional involvement by Albanian physicians, with the potential for both positive and negative consequences. On the one hand, such closeness can foster trust, increase patient satisfaction, and encourage compliance with medical advice. On the other hand, it can place significant emotional stress on physicians, who may feel torn between cultural expectations of relational closeness and their professional limitations including working hours and time to dedicate to their patients. Ultimately, while these culturally embedded relational patterns in Albania may have developed as adaptive responses to weak institutions or limited access to services, they can also pose a significant level of stress to physicians as patients may text or call them permanently even outside regular working hours.

Thus, the lack of clear professional-personal boundaries may lead to an increased accessibility of mobile communication, which, according to evidence, has been identified as a risk factor for stress, sleep disturbances and depression [18]. In this context, we introduce the construct “Work-Related Instant Messaging and Calling (WRIMC) as a measure of the combined exposure to occupational digital communication via messaging and calling across time and settings. Considering all of the above, this study aims to investigate the association between WRIMC and perceived stress among physicians in Albania, positioning it as a potential emerging risk in occupational health.

## Methods

This was a cross-sectional study conducted in Albania during January 2025.

### Study population and sampling

Study population and sampling are described in detail in Appendix 1. Briefly, the study population included 367 participants (response rate: about 90%).

### Measuring instruments and data collection

A structured questionnaire was designed in SurveyMonkey to assess WRIMC stress among physicians in Albania. The questionnaire consisted of 36 items organized into three sections: [1] Demographic and professional characteristics [2], Exposure to WRIMC and associated psycho-physiological factors [3], Perceived Stress Scale (PSS-10) and two additional items regarding the overall perceived impact of work or WRIMC exposure on stress.

The demographic and professional section included 9 items, each operationalized as a variable measured as follows: age (continuous), gender (males vs. females), education level (general practitioner) vs. specialist vs. doctorate vs. postdoctoral), specialty (nominal), residence (categorical), healthcare institutions (categorical), job count (single or part-time job vs. 2 or more jobs), years of experience (continuous), employment status (employed, unemployed, retired, or other), and survey completion context (working during the day, on-call, leisure time, official leave, weekend work, medical leave, or other).

The next section of the questionnaire included 15 items, each operationalized as a variable measured as follows: phone usage frequency for work during a typical workday (never, 1–2 times/day, 3–5 times/day, 6–10 times/day, more than 10 times/day), total time spent on work-related phone use per day (less than 15 min, 15–30 min, 30–60 min, more than 1 h), frequency of receiving WRIMC during a typical workday (never, 1–3 times/day, 4–10 times/day, more than 10 times/day), frequency of receiving WRIMC outside of working hours (never, 1–2 times/day, 3–5 times/day, 6–10 times/day, more than 10 times/day), frequency of using instant messaging apps for work (never, 1–2 times/day, 3–5 times/day, 6–10 times/day, more than 10 times/day), WRIMC frequency during vacations (never, 1–2 times during vacation, 3–5 times, 6–10 times, more than 10 times), WRIMC frequency at night (after 10:00 pm) (never, 1–2 times/month, 1–2 times/week, 3–5 times/week, always), perceived obligation to respond immediately to WRIMC (never, 1–2 times/month, 1–2 times/week, 3–5 times/week vs. always), anxiety when not responding immediately (never, 1–2 times/month, 1–2 times/week, 3–5 times/week, always), interruptions of personal activities due to WRIMC (never, 1–2 times/month, 1–2 times/week, 3–5 times/week, daily), mental fatigue caused by WRIMC (never, 1–2 times/month, 1–2 times/week, 3–5 times/week, daily), irritability due to WRIMC interruptions (never, 1–2 times/month, 1–2 times/week, 3–5 times/week, daily), WRIMC interfering with sleep quality (never, 1–2 times/month, 1–2 times/week, 3–5 times/week, always), persistent work-related thoughts outside office hours (never, 1–2 times/month, 1–2 times/week, 3–5 times/week, daily), and guilt for not responding to WRIMC (never, 1–2 times/month, 1–2 times/week, 3–5 times/week, always).

The third section included 10 items of the PSS-10, an internationally validated questionnaire used to measure stress levels [19], and two additional questions: perceived impact of work on stress level (PIWOSL) (not at all important vs. slightly important vs. moderately important vs. very important vs. extremely important), and perceived impact of WRIMC on stress level (not at all important vs. slightly important vs. moderately important vs. very important vs. extremely important).

The first two sections and the last two items were formulated based on literature research, particularly on techno-stress and occupational stress among healthcare professionals [10, 20–25]. Content validity was assessed through expert review by three specialists in public health and occupational medicine. A pretest was conducted with 20 physicians to assess the clarity, comprehension, and reliability of the questionnaire. Based on their feedback, minor phrasing modifications were made. The questionnaire was distributed via a SurveyMonkey-generated link through physicians’ mobile messaging applications and was self-administered.

Responses to items in the second and third sections were recoded into 4- or 5-point Likert-type scales. Internal consistency, measured by Cronbach’s alpha, was 0.916 for the second section of the instrument and 0.865 for the PSS-10. For the PSS-10, reverse scoring was applied to positively worded items (PSS-4, PSS-5, PSS-7, PSS-8) before computing reliability.

### Data analysis

The PSS-10 score was calculated by reversing the required items and summing all responses, yielding a total score ranging from 0 to 40. Based on established cutoffs, stress levels were categorized as low (0–13), moderate [14–26], and high [19, 27–40].

Frequencies and their respective percentages were calculated for categorical variables, while means and standard deviations were calculated for continuous variables. For ordinal Likert-type items, mean values reflect central tendencies across categories, and not exact counts or durations.

A multivariable-adjusted ordinal logistic regression model was used to assess the direct association between exposure to WRIMC, associated psycho-physiological factors, and perceived stress. A mediation analysis was conducted to examine the indirect effects of WRIMC on perceived stress with the PIWOSL as a mediator. Both crude and multivariable-adjusted models were used to test for potential confounders. Additionally, a moderation analysis was conducted to assess whether the PIWOSL influenced the strength of these associations; however, no significant moderating effect was observed. Other mediators were identified from psycho-physiological factors that showed significant associations with perceived stress in the ordinal logistic regression analysis (specifically irritability, mental fatigue, and guilt related to WRIMC). The mediation analysis included WRIMC exposure factors as independent variables and PSS as the dependent variable. Statistical analyses were conducted using Statistical Package for the Social Sciences (SPSS) version 26, with mediation and moderation analyses performed through the PROCESS v5.0 beta2 macro by Hayes (Model 4), implemented in SPSS.

A p-value of ≤ 0.05 was considered as statistically significant.

## Results

Mean age of study physicians was 42.0 ± 11.3 years; about two-thirds were females; slightly more than half were specialists, followed by general practitioners; around 15% had two or more jobs; approximately 65% completed the survey during their regular workday (Table 1). Most of participants reported moderate stress (62%), and 10% reported high stress. Physicians had 16.6 ± 11.5 years of work experience. Regarding WRIMC exposure, mean values were 3.6 ± 0.7 for daily phone use for work, 3.3 ± 0.9 for total work-related phone time, 2.4 ± 0.7 for WRIMC frequency during the workday, 2.8 ± 1.0 for WRIMC frequency outside work hours, 3.3 ± 0.9 for the use of instant messaging apps for work, 2.8 ± 1.0 for WRIMC during vacations, and 2.0 ± 1.1 for receiving WRIMC after 10:00 pm. A sense of obligation to respond immediately to WRIMC was moderate to high (3.3 ± 0.9), as was feeling anxious when not responding to WRIMC (2.9 ± 1.2), WRIMC interrupting personal activities (2.9 ± 1.0), and WRIMC affecting sleep quality (2.3 ± 1.1). Mental fatigue due to WRIMC was common (2.9 ± 1.0), similar to irritation from WRIMC interruptions (2.5 ± 1.2), persistent work-related thoughts (3.0 ± 1.0), and guilt from not responding to WRIMC (2.5 ± 1.3) (Table 1).

**Table 1:** Descriptive statistics of the study population

Categorical Variables	Frequency (%)
**Gender:**	
Males	125 (34.3)
Females	239 (65.7)
**Educational level**:	
General Practitioner	110 (31.3)
Specialist	187 (53.3)
Doctorate	33 (9.4)
Postdoctoral	21 (6.0)
**Job count**:	
Two or more jobs	57 (15.5)
Single or part-time Job	310 (84.5)
**Survey completion context**:	
Working during the day	234 (64.5)
On-call	32 (8.8)
Leisure time	76 (20.9)
Official leave	3 (0.8)
Weekend work	5 (1.4)
Medical leave	8 (2.2)
Other	5 (1.4)
**Perceived stress levels**:	
Low	97 (27.4)
Moderate	221 (62.4)
High	36 (10.2)

**Numerical Variables**	Mean ± SD^b^
Age *(years)*	42.0 ± 11.3
Work experience *(years)*	16.6 ± 11.5
Work-related phone usage at workplace	3.6 ± 0.7
Work-related total phone usage time	3.3 ± 0.9
Frequency of WRIMC^a^ during workdays	2.4 ± 0.7
WRIMC frequency outside working hours	2.8 ± 1.0
Frequency of work-related use of instant messaging apps (hours)	3.3 ± 0.9
WRIMC frequency during vacations	2.8 ± 1.0
Receiving WRIMC after 10:00 pm	2.0 ± 1.1
Feeling obliged to respond to WRIMC immediately	3.3 ± 0.9
Feeling anxious when not responding to WRIMC	2.9 ± 1.2
WRIMC interrupting personal activities	2.9 ± 1.0
WRIMC affecting sleep quality	2.3 ± 1.1
Mental fatigue due to WRIMC	2.9 ± 1.0
Irritation due to WRIMC interruptions	2.5 ± 1.2
Persistent work thoughts outside office	3.0 ± 1.0
Guilt from not responding to WRIMC	2.5 ± 1.3

^a^WRIMC = Work-related instant messaging and calling

^b^ SD = Standard Deviation

A higher frequency of using instant messaging apps for work-related issues was significantly associated with greater odds of reporting higher stress levels (*P* < 0.01). Irritability due to WRIMC interruptions was the strongest predictor of being in a higher PSS category (*P* < 0.01), while guilt from not responding to WRIMC (*P* < 0.05) and mental fatigue due to WRIMC (*P* < 0.01) also increased the likelihood of transitioning from low or moderate to a higher stress level (Table 2 – Appendix 2).

**Fig. 1:**
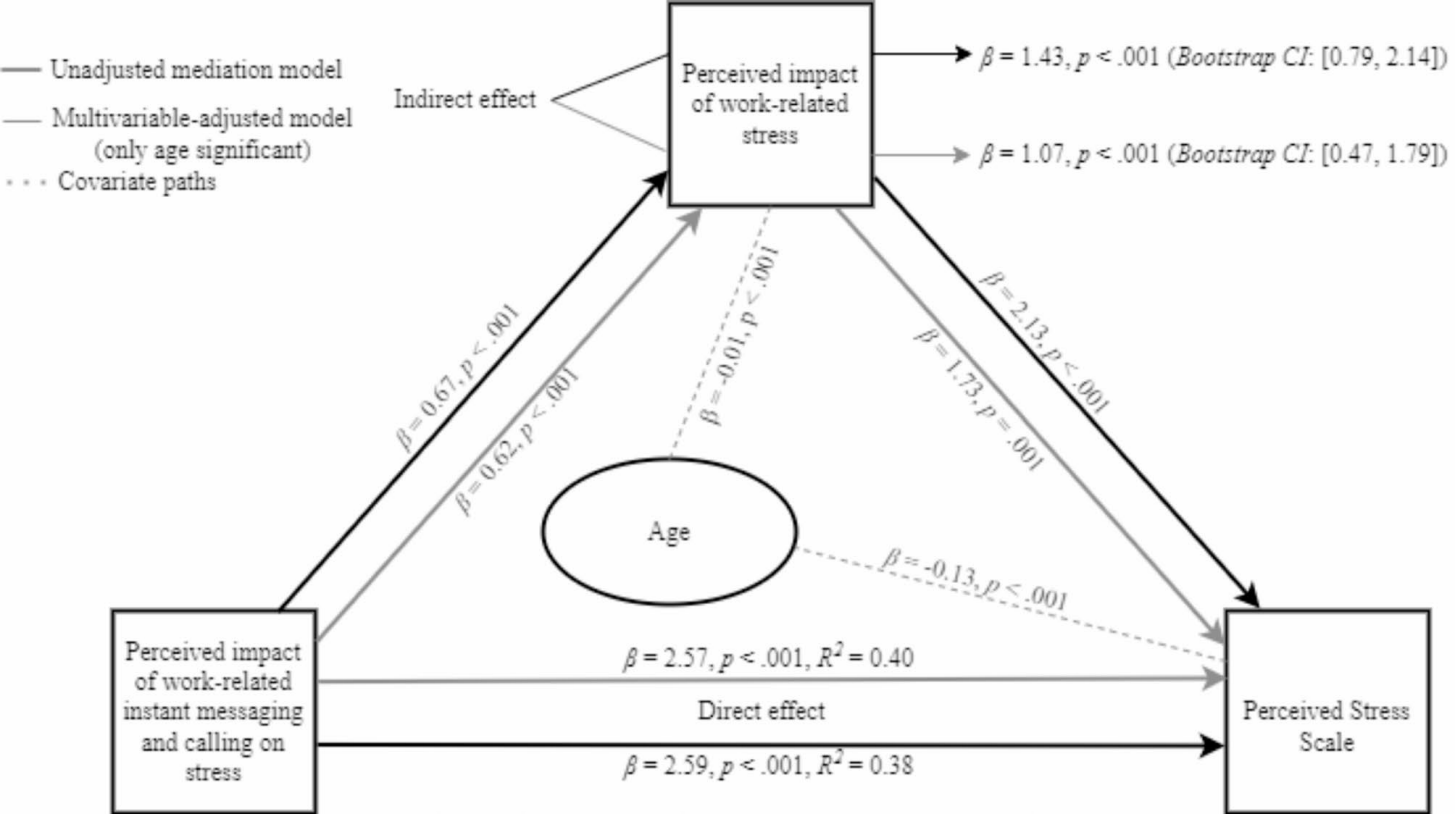
Mediation model of the association between perceived impact of work-related instant messaging and calling on stress and perceived stress scale, with the perceived impact of work-related stress as a mediator

The direct effect of WRIMC on PSS was significant in the unadjusted model (*P* < 0.001) and remained the same in the multivariable-adjusted model (*P* < 0.001). The mediation effect via PIWOSL was significant in the unadjusted model (*P* < 0.001) and remained the same in the multivariable-adjusted model (*P* < 0.001), confirming partial mediation. Age was a significant negative covariate (*P* < 0.001) in the direct effect, and it remained significant in the indirect pathway as well (*P* < 0.001) (Fig. 1).

Irritability due to WRIMC interruptions as (M1) showed full mediation in most of the models, with the strongest effects in work-related phone use frequency during a workday and frequency of receiving WRIMC during a workday, while partially mediated the relationship in the case of receiving WRIMC during vacation (Table 3 – Appendix 2).

Similarly, mental fatigue due to WRIMC frequency (M3) demonstrated full mediation, with the strongest effects observed in work-related phone use frequency during a workday and frequency of receiving WRIMC during a workday. Guilt from not responding to WRIMC (M2) showed the strongest mediating effects in receiving WRIMC during vacations and perceived obligation to respond immediately. On the other hand, guilt from not responding to WRIMC (M2) partially mediated the relationship in total time spent on work-related phone use per day, frequency of receiving WRIMC during a workday, and frequency of using instant messaging apps for work (Table 3 – Appendix 2).

## Discussion

A number of psychological theories attempt to explain the occurrence of occupational stress [26–31]; relatively fewer of them addressing technostress [21, 32], particularly within certain occupational contexts [33], highlighting a gap in quantitative models or studies exploring these issues.

Healthcare professionals frequently report high stress levels, with a study indicating that 83% experienced moderate to high stress [22]. Similarly, in our study, 72.6% of the physicians reported moderate to high stress levels, with 10.2% of them experiencing high stress.

Notably, more than half of physicians experience occupational stress, as evidenced by both a meta-analysis and a study on general practitioners, while burnout prevalence among physicians varies widely from 0 to 80.5% [23, 24, 34]. These variations might be explained by methodological and socio-geographical differences.

Work-related stress among physicians typically arises from a combination of role overload, conflicting responsibilities, bureaucratic demands, excessive workload, and limited autonomy, all contributing significantly to psychological distress and decreased job satisfaction [35]. Given these stressors, it is plausible that the constant exposure to WRIMC may exacerbate feelings of limited control and role ambiguity, further intensifying occupational stress. Building on this, research suggests that frequent digital interruptions in the workplace, such as instant messaging and calling, which are highly salient and difficult to ignore, can increase mental workload perception and contribute to stress [36]. It is noteworthy that such exposures intertwine the occupational context with the broader implications of technology use, including the phenomenon of technostress. Technostress encompasses several elements such as: techno-overload, techno-complexity, techno-invasion, techno-uncertainty and techno-insecurity [21]. Among these, techno-overload and techno-invasion are particularly relevant to physicians’ exposure to WRIMC. For example, a study among nurses in German hospitals identified a significant association between techno-overload and burnout (β = 0.26, *p* < 0.01) [25]. In our study, elements of techno-overload, such as the frequency of using instant messaging apps for work (β = 0.48, *p* = 0.003) and mental fatigue due to WRIMC (β = 0.37, *p* = 0.001), were significantly associated with physicians’ stress levels. In addition to techno-overload, elements of techno-invasion, such as irritability due to WRIMC interruptions (β = 0.65, *p* < 0.001) and guilt from not responding to WRIMC (β = 0.33, *p* < 0.05), are associated with higher stress levels, potentially reflecting blurred work-life boundaries and a perceived expectation of constant availability. Indeed, work-induced guilt has been shown to negatively impact job and life satisfaction, particularly when individuals struggle to manage work demands [37].

In our study, the physicians reported moderate to high exposure to work-related digital communication, with frequent phone use, instant messaging and calling, and a strong obligation to respond immediately. Work-related interruptions occurred beyond work hours, during vacations, and at night, alongside reported experiences of mental fatigue, sleep disturbances, anxiety, guilt, and persistent work-related thoughts. Notably, a scoping review found that smartphone use during off-job hours negatively impacts work-life conflict [38]. Another study highlighted that the physician’s “on-call” status, beyond its impact on work-life conflict, also contributes to sleep disturbances [39]. Aligning with other research evidences [40], our study demonstrated that elements of stress and burnout, such as irritability and mental fatigue, might be related to perceived interruption overload due to WRIMC.

Another important finding is the explanatory role of guilt from not responding to WRIMC, especially the obligation to reply urgently, particularly to instant app messages, which may serve as strong predictors or mediators leading to stress. Actually, guilt has been proven to be a psychophysiological factor that alters sympathetic and parasympathetic activation [41]. Furthermore, the guilt arising from delayed or unanswered responses in mobile instant messaging interactions can exacerbate stress and contribute to emotional exhaustion. Another study has shown that the use of mobile instant messaging for these purposes is linked to feelings of message and information overload, intrusion into personal and face-to-face interactions, pressure to respond promptly, anxiety about receiving timely replies, and confusion regarding the meaning, tone, or intent of messages [42].

While the perceived impact of WRIMC influenced stress directly and indirectly via perceived impact of work-related stress, the role of age suggests that older physicians might experience lower stress levels despite similar exposure (*β* = −0.13, *p* < 0.001). Older physicians consistently report lower psychological distress and burnout, a trend attributed to accumulated experience, professional maturation, and reduced sensitivity to work-related stressors [43, 44]. Also, aging is associated with the development of coping strategies that mitigate technology-related strain, as older workers rely less on behavioral disengagement compared to their younger counterparts [45].

The strongest point of our study was that, for the first time, we established a baseline for the possible impact of WRIMC on stress while considering the partial mediating effect of the occupational context as a medium of exposure and stress, providing a basis for further exploration of factors related to technostress and stress beyond the workplace.

However, this study may have several limitations. Firstly, its cross-sectional nature does not allow the establishment of causal relationships between WRIMC exposure and perceived stress; thus, prospective studies are necessary to draw causal conclusions. Secondly, the self-reported nature of the data may introduce recall bias and social desirability bias, potentially affecting the accuracy of the findings. Thirdly, while internal consistency and content validity of the questionnaire were assessed and showed adequate reliability, further validation of the instrument is needed. Fourthly, despite being under the umbrella of work-related digital communication, instant messaging and calling were not analyzed separately, which limits our ability to assess their distinct effects on perceived stress levels. Finally, given the socio-cultural specificity of Albania, generalizing these findings to other healthcare settings should be done with caution, and future research should explore cross-cultural validation.

## Conclusions

Albanian physicians included in this study reported frequent WRIMC exposure both during and beyond work hours, which was significantly associated with several psycho-physiological stress indicators. Mental fatigue and irritability due to WRIMC interruptions play a crucial role in mediating the relationship between frequent phone usage and WRIMC with perceived stress, while a sense of guilt from not responding to WRIMC primarily mediates the association between frequent WRIMC during vacations and the perceived obligation to respond immediately. Further exploration is needed on the frequency of using instant messaging apps for work, which showed a direct association with perceived stress but may also be linked to other unexplored pathways influencing stress. These findings suggest that the perceived occupational stress context of instant messaging and calling might constitute an important pathway beyond technostress per se, potentially being interconnected with or different from perceived ‘work-related’ stress. Future research should aim to explore causal relationships and propose regulatory and policy-driven interventions to limit WRIMC exposure, thereby mitigating its potential impact on physicians’ well-being.

## Supplementary Information

Additional file 1. Sampling. The sampling frame consisted of the 7,982 physicians listed in the official registry of the Order of Physicians in Albania, representing the target population for this study. The sample size was calculated using the formula for a finite population: n = [N × Z² × p × (1 - p)] / [e² × (N - 1) + Z² × p × (1 - p)] Where: N = 7982 (total number of physicians) Z = 1.96 (95% confidence level) p = 0.5 (assumed proportion) e = 0.05 (desired precision) Thereby, the required sample size was calculated to be 367 participants. In order to account for a 10% expected non-response rate (based on similar assumptions from previous studies conducted in Albania), we determined that sample size be approximately 408 physicians. The sample was drawn using simple random sampling. Of 408 targeted physicians, 32 individuals did not respond, resulting in a response rate of 92.2% (376/408). Additionally, 9 cases with substantial missing data (≥30-40% missing responses) were excluded from the sample. Ultimately, 367 participants were included in the final analysis (final response rate: 367/408≈90%).

Additional file 2. Detailed results pertinent to Table 2 and Table 3.

## Data Availability

No datasets were generated or analysed during the current study.
