# Supplementary material for: Work-related instant messaging and calling stress (WRIMCS) among physicians: a novel occupational health risk?
Source: J Occup Med Toxicol. 2025 Sep 2;20:28. doi: 10.1186/s12995-025-00478-1 (PMC12403839; doi:10.1186/s12995-025-00478-1)
Supplement: Supplementary file 1 — Additional file 1. Sampling. The sampling frame consisted of the 7,982 physicians listed in the official registry of the Order of Physicians in Albania, representing the target population for this study. The sample size was calculated using the formula for a finite population: n = [N × Z² × p × (1 - p)] / [e² × (N - 1) + Z² × p × (1 - p)] Where: N = 7982 (total number of physicians) Z = 1.96 (95% confidence level) p = 0.5 (assumed proportion) e = 0.05 (desired precision) Thereby, the required sample size was calculated to be 367 participants. In order to account for a 10% expected non-response rate (based on similar assumptions from previous studies conducted in Albania), we determined that sample size be approximately 408 physicians. The sample was drawn using simple random sampling. Of 408 targeted physicians, 32 individuals did not respond, resulting in a response rate of 92.2% (376/408). Additionally, 9 cases with substantial missing data (≥30-40% missing responses) were excluded from the sample. Ultimately, 367 participants were included in the final analysis (final response rate: 367/408≈90%). [file 12995_2025_478_MOESM1_ESM.docx]

**Additional file 1. Sampling**

The sampling frame consisted of the 7,982 physicians listed in the official registry of the Order of Physicians in Albania, representing the target population for this study.

The sample size was calculated using the formula for a finite population:

n = [N × Z² × p × (1 - p)] / [e² × (N - 1) + Z² × p × (1 - p)]

Where:

N = 7982 (total number of physicians)

Z = 1.96 (95% confidence level)

p = 0.5 (assumed proportion)

e = 0.05 (desired precision)

Thereby, the required sample size was calculated to be 367 participants.

In order to account for a 10% expected non-response rate (based on similar assumptions from previous studies conducted in Albania), we determined that sample size be approximately 408 physicians. The sample was drawn using simple random sampling.

Of 408 targeted physicians, 32 individuals did not respond, resulting in a response rate of 92.2% (376/408). Additionally, 9 cases with substantial missing data (≥30-40% missing responses) were excluded from the sample. Ultimately, 367 participants were included in the final analysis (final response rate: 367/408≈90%).
