# Supplementary material for: Work-related instant messaging and calling stress (WRIMCS) among physicians: a novel occupational health risk?
Source: J Occup Med Toxicol. 2025 Sep 2;20:28. doi: 10.1186/s12995-025-00478-1 (PMC12403839; doi:10.1186/s12995-025-00478-1)
Supplement: Supplementary file 2 — Additional file 2. Detailed results pertinent to Table 2 and Table 3. [file 12995_2025_478_MOESM2_ESM.docx]

**Additional file 2. Detailed results pertinent to Table 2 and Table 3**

**Table 2. Ordinal logistic regression analysis of WRIMC exposure and associated psycho-physiological factors predicting perceived stress among physicians**

| **Predictors (independent variables)** | **Estimate (β)** | **SE**^†^ | **Wald** | **P^*^** | **95% CI^**^** |
| --- | --- | --- | --- | --- | --- |
| **WRIMC**‡**’s exposure** |  |  |  |  |  |
| Frequency of using instant messaging apps for work | 0.48 | 0.16 | 8.69 | 0.003 | 0.16–0.79 |
| **Psychophysiological factors** |  |  |  |  |  |
| Irritability due to WRIMC interruptions | 0.65 | 0.18 | 13.35 | <0.001 | 0.30–0.99 |
| Guilt from not responding to WRIMC | 0.33 | 0.15 | 4.69 | 0.03 | 0.03–0.62 |
| Mental fatigue due to WRIMC | 0.37 | 0.12 | 10.26 | 0.001 | 0.14–0.60 |

**^*^**P-value <0.05 indicates statistical significance. Only statistically significant associations are reported.
**^†^** **SE** = Standard Error.
**^**^** **95% CI** = 95% Confidence Interval.
**^‡^** **WRIMC** = Work-related instant messaging and calling.

**Table 3. Mediating effects of WRPPSF between WRIMC and Perceived Stress Score**

| **Path Model X1^‡^ → M^§^ → Y^¶^** | **Direct Effect  (β, p-value)** | **Indirect Effect  (β, Boot CI^*^)** | **Mediation Type** | **R²**^†^ |
| --- | --- | --- | --- | --- |
| X1 → M1 → Y | -0.45, p = 0.351 | 2.03 (1.46 - 2.67) | full | 0.26 |
| X1 → M2 → Y | 0.88, p = 0.076 | 0.69 (0.31 - 1.12) | full | 0.13 |
| X1 → M3 → Y | -0.65, p = 0.174 | 2.23 (1.49 - 3.08) | full | 0.29 |
| X2 → M1 → Y | 0.07, p = 0.846 | 1.52 (1.06 - 2.01) | full | 0.26 |
| X2 → M2 → Y | 0.98, p = 0.011 | 0.60 (0.30 - 0.97) | partial | 0.14 |
| X2 → M3 → Y | 0.11, p = 0.757 | 1.48 (1.00 - 2.02) | full | 0.29 |
| X3 → M1 → Y | -0.61, p = 0.229 | 2.27 (1.63 - 2.93) | full | 0.26 |
| X3 → M2 → Y | 1.12, p = 0.025 | 0.52 (0.14 - 0.97) | partial | 0.14 |
| X3 → M3 → Y | -0.42, p = 0.381 | 2.08 (1.38 - 2.84) | full | 0.29 |
| X4 → M1 → Y | -0.13, p = 0.713 | 1.49 (1.06 - 1.97) | full | 0.26 |
| X4 → M2 → Y | 0.62, p = 0.106 | 0.73 (0.40 - 1.11) | full | 0.13 |
| X4 → M3 → Y | -0.06, p = 0.866 | 1.42 (0.94 - 1.96) | full | 0.29 |
| X5 → M1 → Y | 0.06, p = 0.885 | 1.67 (1.19 - 2.20) | full | 0.25 |
| X5 → M2 → Y | 1.14, p = 0.003 | 0.58 (0.26 - 0.99) | partial | 0.14 |
| X5 → M3 → Y | 0.19, p = 0.608 | 1.53 (1.02 - 2.15) | full | 0.29 |
| X6 → M1 → Y | -0.79, p = 0.032 | 1.82 (1.32 - 2.37) | partial | 0.26 |
| X6 → M2 → Y | 0.22, p = 0.560 | 0.79 (0.46 - 1.18) | full | 0.12 |
| X6 → M3 → Y | -0.61, p = 0.083 | 1.63 (1.16 - 2.16) | full | 0.29 |
| X7 → M1 → Y | -0.31, p = 0.337 | 1.33 (0.93 - 1.77) | full | 0.26 |
| X7 → M2 → Y | 0.42, p = 0.214 | 0.59 (0.30 - 0.92) | full | 0.13 |
| X7 → M3 → Y | -0.16, p = 0.605 | 1.18 (0.76 - 1.65) | full | 0.29 |
| X8 → M1 → Y | -0.34, p = 0.354 | 0.97 (0.54 - 1.45) | full | 0.26 |
| X8 → M2 → Y | -0.21, p = 0.601 | 0.83 (0.47 - 1.26) | full | 0.12 |
| X8 → M3 → Y | -0.60, p = 0.096 | 1.24 (0.71 - 1.77) | full | 0.29 |

**^*^Boot CI** = Bootstrapped confidence interval (5,000 resamples).
**^†^R²** = Proportion of variance explained by the model.
**^‡^**Independent variables (X1–X8): Frequency of work-related phone use (X1), total time spent on work-related phone use (X2), frequency of work-related instant messaging and calling during work hours (X3), outside work hours (X4), using instant messaging apps (X5), during vacations (X6), after 10:00 pm (X7), and perceived obligation to respond immediately (X8).
**^§^**Mediating variables (M1–M3): Irritability due to work-related instant messaging and calling (M1), guilt from not responding to work-related instant messaging and calling (M2), and mental fatigue due to work-related instant messaging and calling (M3).
**^¶^**Dependent variable: Perceived stress score.
